# The Actin Associated Protein Palladin Is Important for the Early Smooth Muscle Cell Differentiation

**DOI:** 10.1371/journal.pone.0012823

**Published:** 2010-09-22

**Authors:** Li Jin, Qiong Gan, Bartosz J. Zieba, Silvia M. Goicoechea, Gary K. Owens, Carol A. Otey, Avril V. Somlyo

**Affiliations:** Department of Molecular Physiology and Biological Physics, University of Virginia, Charlottesville, Virginia, United States of America; Department of Cell and Molecular Physiology, University of North Carolina at Chapel Hill, Chapel Hill, North Carolina, United States of America; Cleveland Clinic, United States of America

## Abstract

Palladin, an actin associated protein, plays a significant role in regulating cell adhesion and cell motility. Palladin is important for development, as knockdown in mice is embryonic lethal, yet its role in the development of the vasculature is unknown. We have shown that palladin is essential for the expression of smooth muscle cells (SMC) marker genes and force development in response to agonist stimulation in palladin deficient SMCs. The goal of the study was to determine the molecular mechanisms underlying palladin's ability to regulate the expression of SMC marker genes. Results showed that palladin expression was rapidly induced in an A404 cell line upon retinoic acid (RA) induced differentiation. Suppression of palladin expression with siRNAs inhibited the expression of RA induced SMC differentiation genes, SM α-actin (SMA) and SM22, whereas over-expression of palladin induced SMC gene expression. Chromatin immunoprecipitation assays provided evidence that palladin bound to SMC genes, whereas co-immunoprecipitation assays also showed binding of palladin to myocardin related transcription factors (MRTFs). Endogenous palladin was imaged in the nucleus, increased with leptomycin treatment and the carboxyl-termini of palladin co-localized with MRTFs in the nucleus. Results support a model wherein palladin contributes to SMC differentiation through regulation of CArG-SRF-MRTF dependent transcription of SMC marker genes and as previously published, also through actin dynamics. Finally, in E11.5 palladin null mouse embryos, the expression of SMA and SM22 mRNA and protein is decreased in the vessel wall. Taken together, our findings suggest that palladin plays a key role in the differentiation of SMCs in the developing vasculature.

## Introduction

Vascular smooth muscle cells (SMCs) are not terminally differentiated. They have the ability to undergo phenotypic switching in association with pathological conditions such as vascular injury, post angioplasty stenosis, and atherosclerosis. Phenotypic plasticity of SMCs is critical for the establishment of a mature vessel, which can function to regulate vascular tone and blood vessel diameter, peripheral resistance, and the distribution of blood flow throughout the developing organism. Considerable evidence suggests that an impaired SMC phenotype during development results in defects in vascular remodeling of great arteries and congenital cardiovascular anomalies, but a full understanding of the complex processes underlying SMC development is still elusive.

The differentiation of SMC is characterized by the up-regulation of SMC marker genes, which are associated with the contractile phenotype, such as SM alpha actin (SMA), SM myosin heavy chain (MHC) and SM22. The expression of SMC marker genes has been shown to be regulated by CArG-SRF complexes, by myocardin and by Myocardin Related Transcription Factors MRTF-A and MRTF-B that induce transcription of SMC marker genes in a CArG dependent manner [1], [2], [3], [4], [5]. Other factors, including Elk-1, Foxo4, and KLF4 [4], [5], [6], [7] have been identified as repressors for SMC marker gene expression. The actin cytoskeleton is both an upstream regulator of MRTF activity, with monomeric (G) actin directly acting as a signal transducer, and a downstream effector of SRF resulting in activation of cluster genes encoding components of the actin cytoskeleton [8], [9], [10].

Actin dynamics plays an important role in regulation of SRF mediated transcription of SMC marker genes [2], [11]. Rho signaling or other stimuli that promote actin polymerization determine the availability of G actin. A decrease in the G actin pool is both necessary and sufficient for SRF to activate expression of SMC genes. G actin also shuttles into and out of the nucleus, where it is thought to regulate chromatin structure and transcription[12]. G actin sequesters MRTFs in the cytoplasm by binding to the MRTF amino-terminal RPEL domain thereby inhibiting MRTF nuclear import, nuclear accumulation, and SRF-mediated transcription. FRET experiments have shown that MRTF and actin interact both in the cytoplasm and in the nucleus, and that this interaction is the downstream of RhoA-mediated changes in actin turnover [12].

The actin associated protein palladin is a widely expressed protein found in stress fibers, focal adhesions, podosomes, dorsal ruffles, growth cones, Z-discs, and other actin-based subcellular structures [13]. Palladin is required for the maintenance of normal stress fibers in cultured cells [14], [15]. It belongs to a small gene family that includes the Z-disc proteins myopalladin and myotilin, all of which share similar Ig-like domains [13], [16], [17], [18]. Other palladin family members expressed in skeletal muscle are important for sarcomere integrity and mutated forms are associated with inherited muscular disorders [13], [19]. Palladin serves as a scaffold for multiple actin binding proteins, signaling molecules, and also as an actin cross-linking protein [20]. Knockdown of palladin causes decreased F to G actin ratio, faint and disordered stress fibers in cultured fibroblasts and SMCs [15], [21], [22]. Overexpression of palladin induces strong stress fibers in cultured cells[23]. Palladin is highly expressed in smooth muscle and we and others have previously shown it is important in organization of the SMC cytoskeleton and in regulating contraction [24], [25]. Moreover, we recently published results showing that palladin knockout embryonic stem cells (ESC) exhibit impaired differentiation into SMC in a embryoid body (EB) SMC differentiation model system [26]. Knock out of palladin in mice is embryonic lethal at day 15.5[27]. However, it is unknown if palladin KO mice show impaired differentiation of SMC, and little is known regarding the cellular and molecular mechanisms by which palladin contributes to control of SMC differentiation.

The present studies focused on determining the role of palladin in early SMC differentiation and vasculature development. Results showed that palladin expression was rapidly induced during the differentiation of SMCs, and induces expression of multiple SMC differentiation marker genes by interacting with the MRTF complexes, which directly or indirectly bind to the CArG elements in the promoter regions of SMC marker genes. Moreover, E11.5 palladin KO mouse embryos exhibited markedly impaired differentiation of vascular SMC. This study provides the first evidence that the cytoskeletal protein palladin plays a critical role in the regulation of early stages of SMC differentiation.

## Materials and Methods

We certify that all research using vertebrate animals in the grant application above is contained in the protocol listed. We certify that all research using cell lines in the grant application above is contained in the protocol listed. Protocol entitled “studies of Signal Transduction in Smooth and Cardiac Muscles in Genetically Altered Mice” (protocol No 2796) is approved by University of Virginia animal care and use committee; The protocol title for the knockout mice is “Exploring the role of palladin in anterior neural tube closure,” and the number 10-039.0. This protocol is approved by University of North Carolina at Chapel Hill Animal care and use committee.

### Cell culture, transient transfection and luciferase assay

A404 cells were cultured and induced to differentiate to SMCs as described previously [26], [28]. Wild type, palladin knockout, and myocardin knockout SMC like cells APSCs (SMA-puromycin-selected-cells) derived from embryonic stem cells/EBs were cultured as described previously[26], [29]. Transfection of plasmids was performed with lipofectamine 2000 (Invitrogen, CA) following the manufacturer's instruction. Culture of rat aortic SMCs (R518) were described previously, [24], [28]. R518 cells were transfected by electroporation. For nuclear fractionation, sub confluent R518 cells were treated with 20 and 50 nM of leptomycin B (LMB) overnight. Nuclear fractionation was carried out according to the manufacturer's protocol (Pierece, IL). A404 cells were transfected with palladin siRNA oligos (Darmacon, NC) using oligofectamine (Invitrogen, CA) according to the manufacturer's protocols. Cells were transfected with 100–300 nM siRNA oligo for 6 h before the addition of all-trans retinoic acid (RA) (Sigma, MO). KLF4, ACLP (aortic carboxypeptidase-like protein), SMA and SM22 luciferase constructs and its CArG mutants were described previously [30], [31], [32]. Luciferase activity was measured and normalized to the total protein content. MRTF RPEL motif mutant constructs (the mut1 mutated R33A and P34A; the mut2 mutated L39A and V40A) were made with the QuickChange site directed mutagenesis kit according to the supplier's instructions (Stratagen).

### Real-Time RT-PCR

Total RNA was extracted with Trizol regent and cDNA was generated with a cDNA synthesis kit as previously described. Quantitative RT-PCR was performed as published [28], [29], [33] using specific primers and probes.

### In vitro translation and GST pulldown assays

Flag tagged MRTF A/B[30] proteins were translated in TnT T7 quick coupled transcription/translation system (Promega, WI). GST tagged palladin and its truncated mutants were described previously [24]. GST-palladin-glutathione sepharose beads were incubated with flag tagged MRTF A/B protein for 2 h at 4°C and washed three times. Bead bound proteins were eluted by boiling and analyzed with Transcend™ non-radioactive translation detection systems following the manufacturer's instructions (Promega, WI).

### Western blot

Tissues or cultured cells were lysed in RIPA buffer supplemented with 1% of proteinase inhibitor cocktail. The extracts were cleared at 15,000 g for 10 min at 4°C. Nucleus fractionation was performed according to the manufacture's protocol (Pierece, IL). The equal amounts of protein were subjected to SDS-PAGE. Western blotting was performed as published methods [24], [28]. The sources of antibody used: palladin polyclonal antibody generated in rabbit recognizes the 140 and 90 kDa palladin isoforms based on Western blotting, SMA monoclonal antibody (Sigma, MO), SM22 polyclonal antibody (Abcam, MA), GAPDH monoclonal antibody (Millipore, MA). All other antibodies are from Santa Cruz Biotechnology (Santa Cruz, CA). The results were analyzed on an ODYSSEY infrared imaging system.

### ChIP assay

ChIP assays were performed as previously described [32]. A404 cells were treated with RA (1 µmol) for 48 h. A404 and R518 cells were fixed with 1% formaldehyde for 10 min at 37°C to cross-link protein-DNA and protein–protein interactions within intact chromatin. Cells were harvested and sonicated to shear chromatin fragments to 200–600 base pairs. Chromatin-protein complexes were immunoprecipitated with antibodies against palladin and SRF. Salmon sperm DNA and protein A agarose beads were added to the complexes. Samples were washed and reversibly cross linked. Recovered DNA was quantified by fluorescence with picogreen reagent (molecular probes, OR). Real time PCR was performed to amplify the CArG containing region of the SMA promoters [32].

### Generation of palladin −/− mice

See supplemental methods Text S1 and Figure S1.

### Indirect Immunofluorescence

Cultured cells were fixed with cold methanol or 4% paraformaldehyde and permeabilized with 0.03% Triton X-100 in PBS rinsed and blocked for 1 h in PBS containing 3% BSA and then incubated with an anti-myc monoclonal antibody (1∶1000), anti-palladin polyclonal antibody (1∶800), anti-MRTF polyclonal antibody (1∶200) diluted in blocking solution for 2 h at RT or overnight at 4°C. Secondary antibodies were Alexa 594 conjugated goat anti-rabbit IgG (1∶1000; Molecular Probes) and Alexa488 goat anti-mouse IgG (1∶1000) in blocking buffer (Jackson ImmunoResearch, PA). Cells were washed four times for 5 min with PBS before being mounted with Aqua Poly/Mount (Polysciences, PA). Confocal images were obtained on an Olympus FV300 microscope.

#### Immunohistochemistry

Paraffin embedded embryo frontal sections (5 µm thick) were treated and stained as previous described [28], [34], [35]. Primary antibodies were: rabbit anti-palladin polyclonal antibody (1∶1500 dilution), anti-SMA monoclonal antibody conjugated with alkaline phosphatase (1∶800 dilution, Sigma, MO); rabbit SM22 polyclonal antibody (1∶500 dilution, Abcam, MA).

## Results

### Palladin was induced in SMC differentiation model

**Figure 1:**
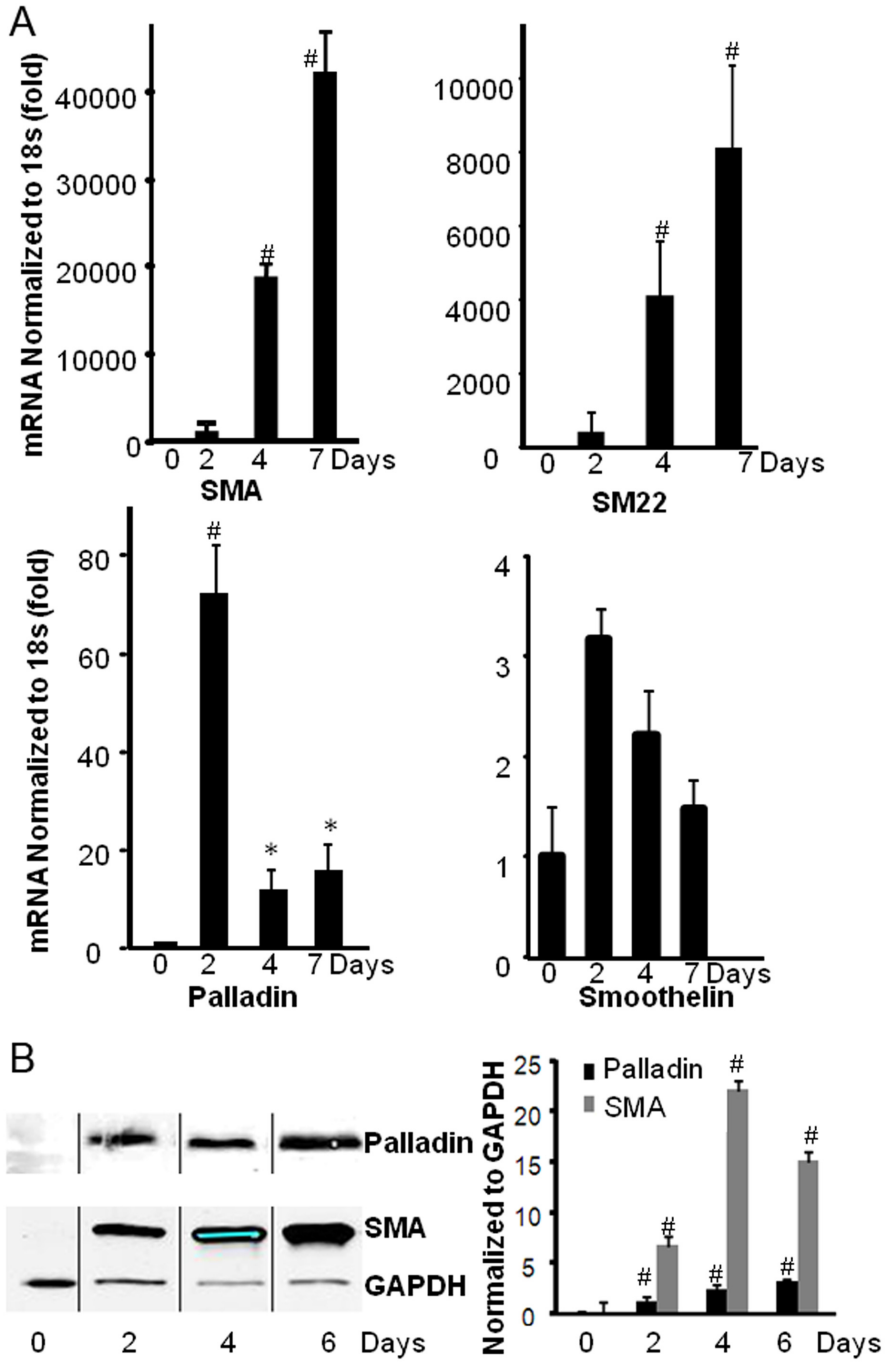
the expression of palladin, SMA, and SM22 is induced during SMC differentiation. A. Undifferentiated A404 cells were induced to differentiate into SMCs by treatment with RA (1 µmol/L). Cells were harvested at different time points, and mRNA was isolated. Palladin and SMC marker genes SMA, SM22, and smoothelin were quantified with real time RT-PCR, and normalized to ribosomal18s. Values represent the mean ±SEM of three independent experiments (n = 3). # p<0.01; * p<0.05 B. Representative Western blots show that SMA and palladin are induced at the protein level in A404 cells treated with RA. Undifferentiated subconfluent A404 cells were treated with RA, and cells were lysed with RIPA buffer at different time points. An equal amount of protein was loaded for SDS-PAGE, and blotted with anti-SMA, palladin, and GAPDH antibodies. The protein expression was normalized to GAPDH. Quantification of protein expression is showing in the bar graph. Values represent the mean ±SEM (n = 3). # p<0.01.

The importance of palladin in SMC differentiation was examined in a clonal line of SMC progenitor cells designated A404 cells [36]. The A404 cells were developed from P19 mouse embryonic carcinoma cells transfected with a SMA promoter/puromycin-N-acetyltransferase [36]. This cell line can be induced to coordinately activate all known SMC differentiation marker genes by treatment with all-trans retinoic acid (RA) [36]. Results of the present studies showed a 70-fold increase in mRNA expression of the 92 kDa isoform of palladin 48 hrs after RA treatment (Fig. 1, p<0.05). These results are consistent with our previous findings showing induction of palladin expression within ESC-EBs treated with RA [26]. In both A404 cells and the ESC-EB model, following the initial induction of palladin, preceeding SMC marker gene expression, there was not a direct correlation between the level of palladin mRNA and SMA mRNA. This may reflect regulation of mRNA through degradation or translation. On the other hand, both palladin and SMA protein expression continued to increase through days 2 through 6.

### Palladin is both necessary and sufficient to induce expression of multiple CArG-dependent SMC marker genes in A404 progenitor cells

**Figure 2:**
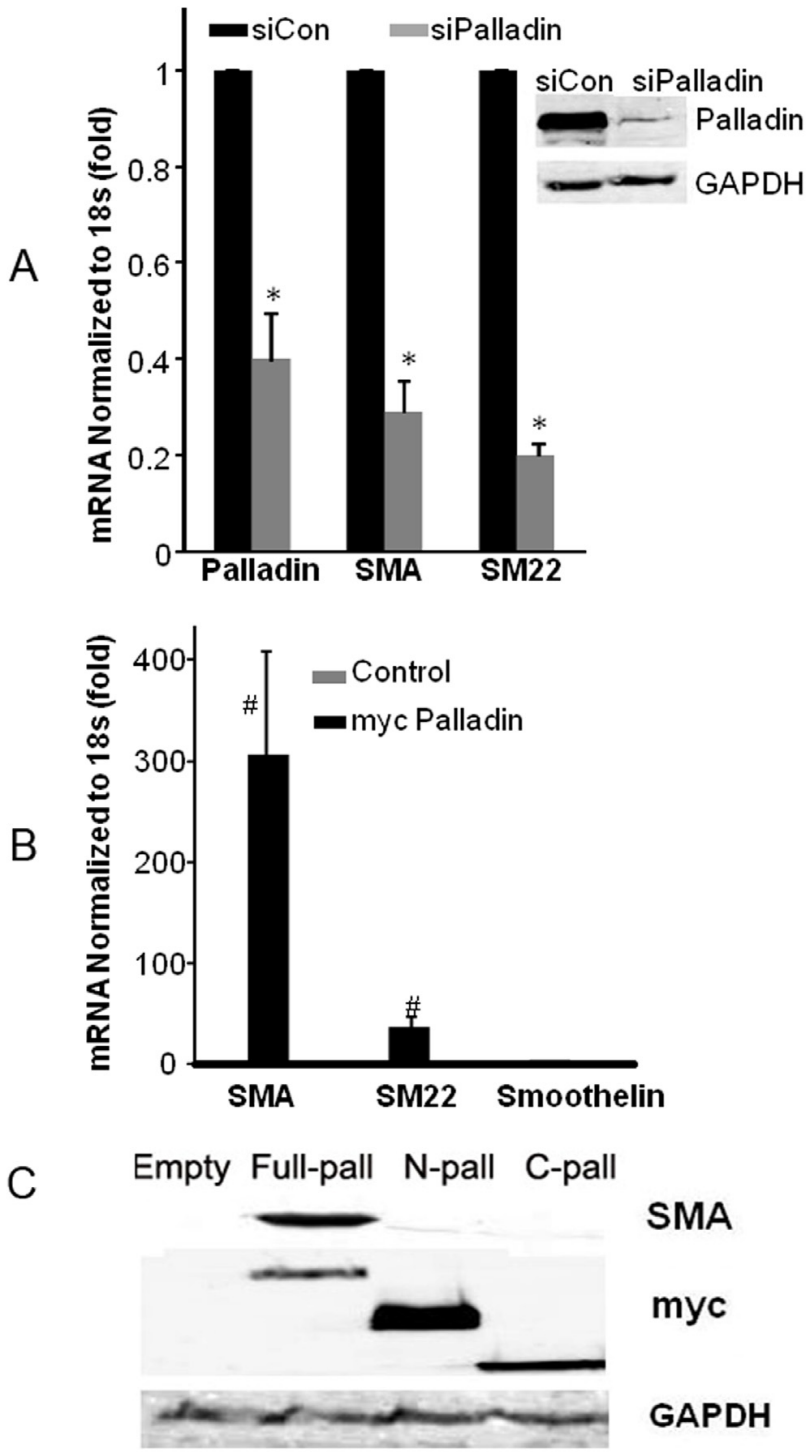
SMA and SM22 genes are down or up regulated by silencing or overexpression of palladin. A) Undifferentiated A404 cells were transfected with siRNA oligos and then treated with RA for 48 h. The expression of SMA, SM22, and palladin was determined by real time RT-PCR, and normalized to ribosomal 18s. Values represent the mean±SEM, n = 3. * p<0.05. Inset shows overexpressed palladin was knocked down with siRNA. B) Undifferentiated A404 cells were transfected with myc tagged palladin constructs, and cells were harvested 36 h after transfection. The mRNA expression of SMC marker genes was measured by real time RT-PCR using specific primers, and normalized to ribosomal18s. Values represent the mean±SEM (n = 3). #,p<0.01. C) Representative Western blots show that overexpression of full length, but not the amino (N) or carboxyl (C) terminal halves of palladin can induce SMA expression. Smoothelin expression was not changed. Undifferentiated A404 cells were transfected with myc tagged palladin constructs, and harvested 36 h after transfection. The cell lysates were loaded for SDS-PAGE, and blotted with anti-SMA, palladin, and GAPDH antibodies. The protein expression was normalized to GAPDH.

To determine if palladin induces the expression of endogenous SMC differentiation marker genes, myc tagged palladin expression constructs were transfected into undifferentiated A404 cells. As shown in Fig. 2B and 2C, the expression of SMA and SM22 was significantly increased at both the mRNA level and protein levels. However, in contrast, there was no induction of the CArG independent SMC marker gene smoothelin. Induction of CArG-dependent SMC marker genes required full length palladin as neither the amino- or carboxyl-terminal deletion mutants of palladin were effective (Fig. 2C, p<0.01). Conversely, siRNA induced suppression of palladin was associated with a 70–80% reduction in SMA and SM22 expression in RA treated A404 cells (Fig. 2A, p<0.05), but no change in smoothelin expression (data not shown). This effect is particularly impressive given that the palladin siRNA only suppressed palladin expression by approximately 60% in these experiments. Of interest, siRNA induced suppression of palladin was not associated with any change in expression of myocardin and MRTFs A and B (data not shown) indicating effects are not secondary to reduced expression of these potent CArG-SRF co-activators.

### Palladin induced SMC marker genes through CArG-dependent mechanisms

**Figure 3:**
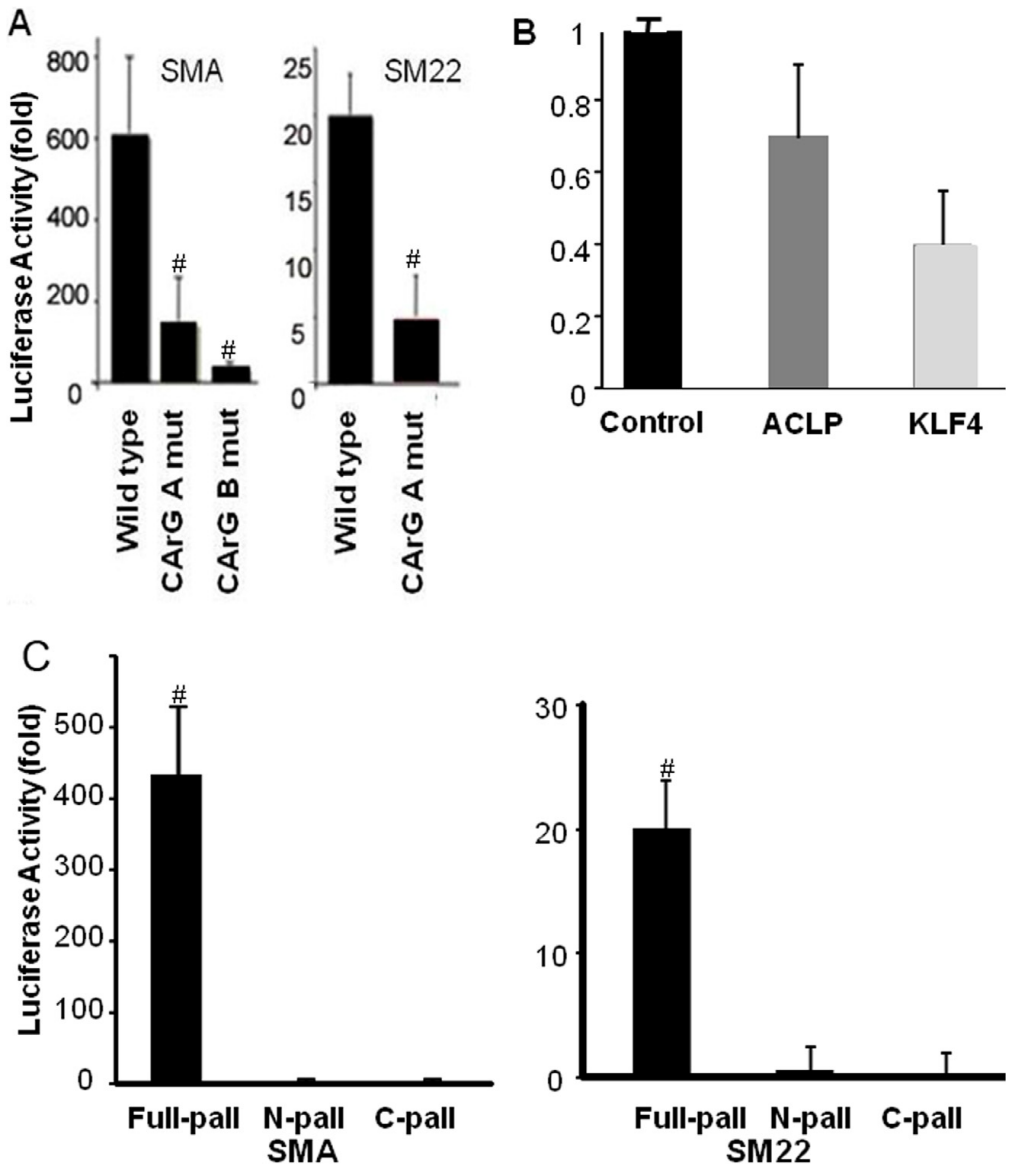
Palladin induced SMC marker genes transcription is mediated in part through the CArG elements found within the SM22 and SMA promoters. SM22 and SMA promoter-enhancer-luciferase plasmids were co-transfected with a palladin expression plasmid into undifferentiated A404 cells. Cells were lysed 36 h after transfection, and luciferase assays were performed and normalized to protein contents. A. Palladin significantly induces the transcriptional activity of wild type SMA and SM22. Mutation of CArG in SM22 or CArG A (A) and B (B) in the SMA promoter decreased the response to palladin (p<0.01 compared to wild type; n = 3). B) Palladin had no significant effect on the transcriptional activity of the CArG independent gene, ACLP, while KLF4 activity decreased (n = 3, p>0.05). C) Full length palladin, but neither amino (N) or carboxyl (C) termini of palladin, induced SMC gene transcriptional activity. Values represent the mean±SEM (n = 3). #, p<0.01.

Transient transfection studies with wild type and CArG mutant SM22 and SMA promoter-enhancer-luciferase plasmids were done to determine if palladin induces expression of SMC marker genes through CArG-dependent mechanisms. Luciferase activity results showed that palladin induced the transcriptional activity of wild type SMA and SM22 promoter-enhancer-luciferase constructs by 600 and 20-fold respectively in A404 cells (Fig. 3A, p<0.01). Full-length palladin is necessary for the induction of SMC marker genes promoter activity, as either amino terminal or carboxyl terminal half alone cannot induce this activity (Figure 3C, p<0.01). In contrast, the CArG mutant promoters SMA and SM22 were not activated as compared to the wild type constructs (Fig. 3A, p<0.01). These results suggest that palladin-dependent induction of SMC marker genes was mediated in part through the CArG-SRF complex. Luciferase reporter assays showed that the promoter activity of the CArG independent ACLP gene was not changed, whereas the KLF4 promoter activity was decreased (Figure 3B).

**Figure 4:**
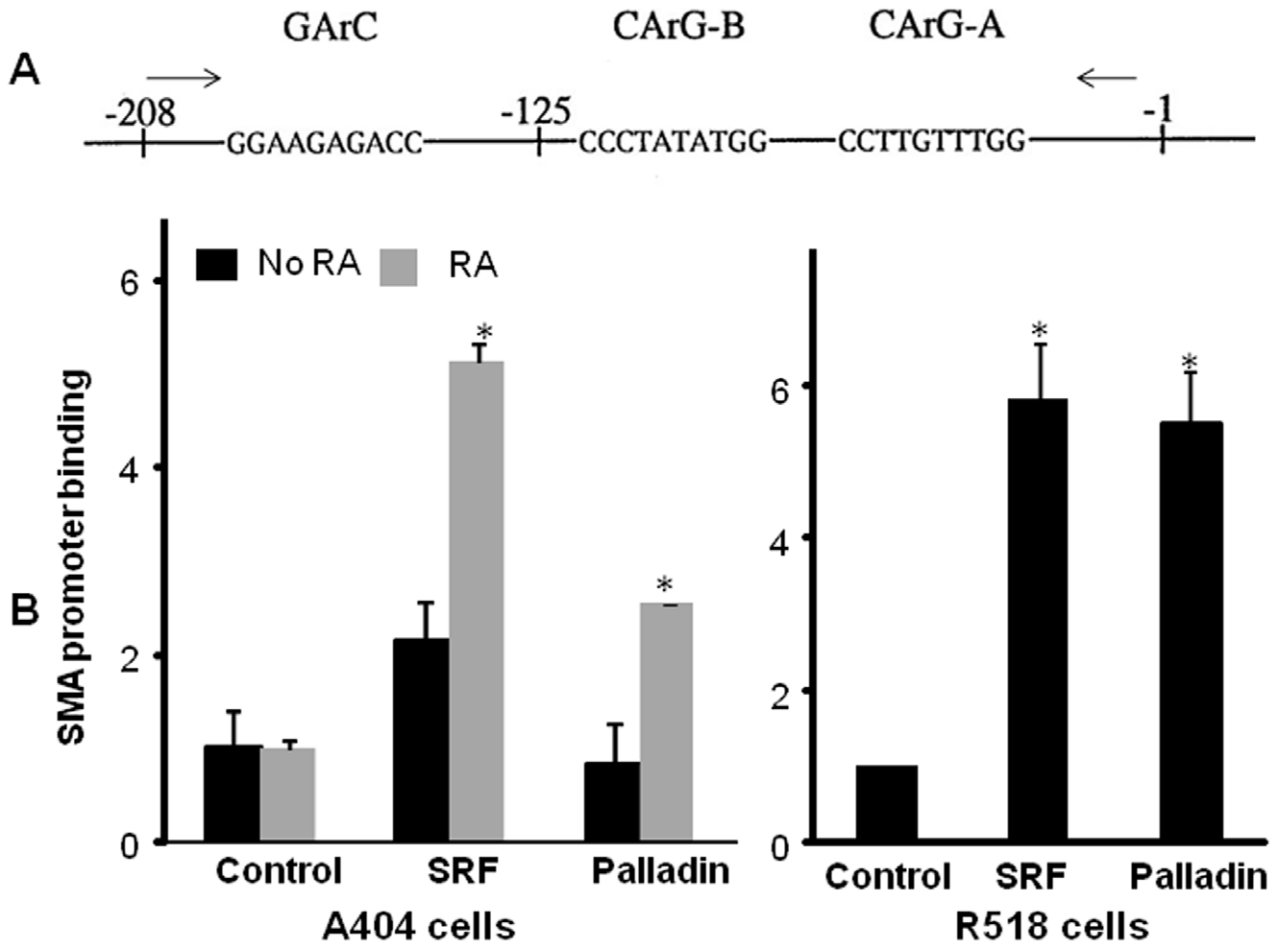
Chromatin immunoprecipitation assay showing that palladin associates with the SMA promoter in differentiated A404 cells and rat aortic SMCs. A) Schematic structure showing the SMA promoter region amplified in ChIP assay. B) Undifferentiated A404 cells were differentiated into SMCs with RA. A404 cells were harvested 48 h after RA treatment, and chromatin IP was performed. Subconfluent R518 cells under normal culture condition were harvested and ChIP assays performed with SRF and palladin antibodies. The intensity of SMA promoter was quantified with real time PCR. No antibody was used as negative control. Values represent the mean±SEM (n = 3). *, p<0.05.

Binding of endogenous palladin to the SMA promoter was also examined in A404 and rat aortic SMCs using ChIP assays. A404 cells were differentiated into SMCs with RA for 48 h, and harvested for ChIP assays. As shown in Fig. 4, there was marked enrichment of palladin binding to the endogenous SMA promoter region in both A404 and R518 cells (p<0.05).

### Palladin, MRTF and Myocardin were interdependent in regulation of SMC genes

**Figure 5:**
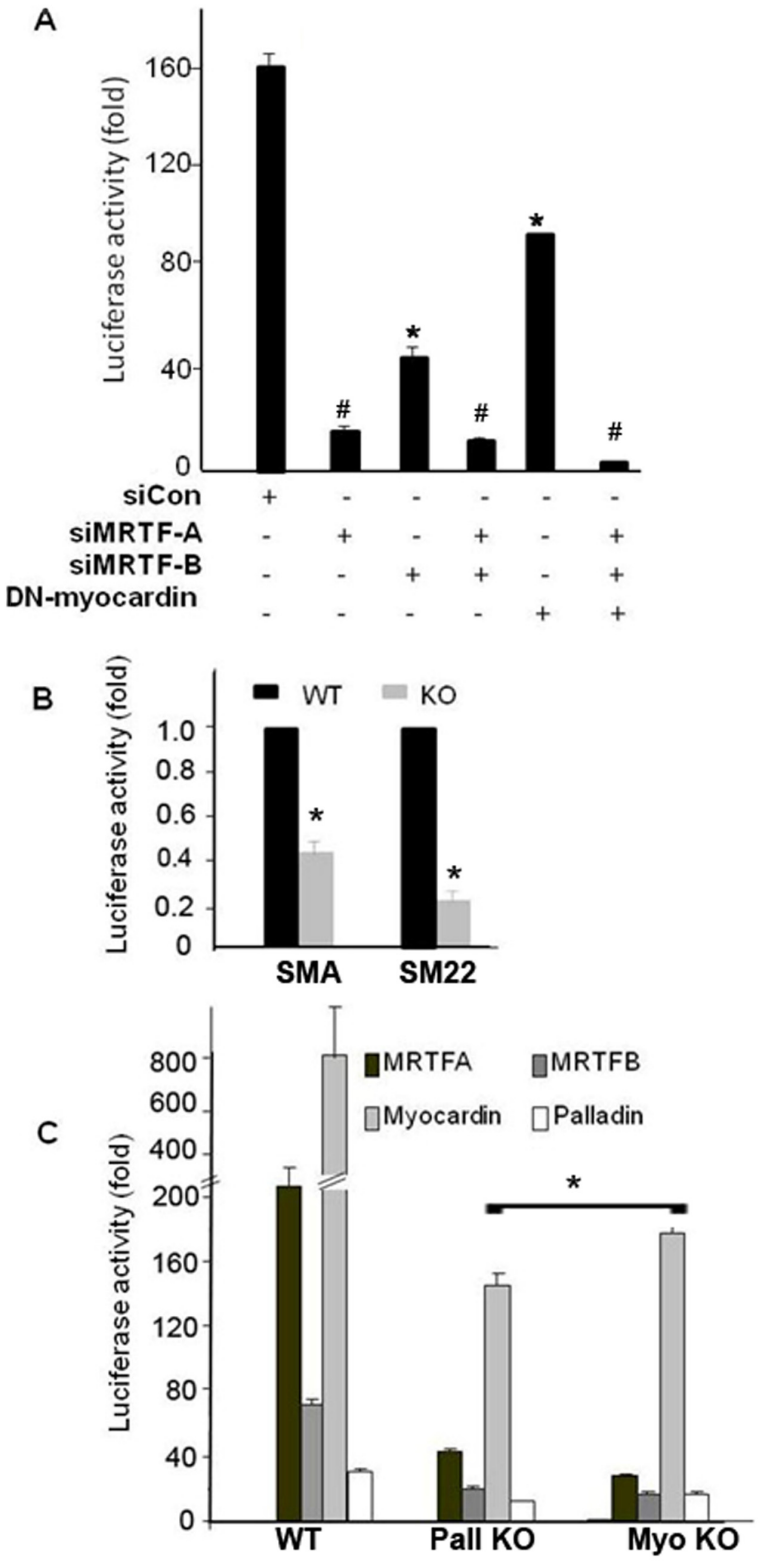
Palladin induces SMC marker gene transcription through the myocardin-MRTF-SRF pathway. A) down regulation of MRTFs and myocardin attenuates palladin induced SMA activity in A404 cells. siRNA to MRTFs (siMRTF-A and siMRTF-B) and dominant negative myocardin (DN Myo) constructs were cotransfected with SMA promoter luciferase constructs alone or with a palladin expressing plasmid into A404 cells, and luciferase activities were measured. Values represent the mean±SEM (n = 3). *, p<0.05; # p<0.01. B) SMA and SM22 promotor transcriptional activities were decreased in palladin null APSCs. Wild type and palladin null APSCs were transfected with SMA and SM22 luciferase constructs. Luciferase assays were performed 36 h after transfection. Values represent the mean±SEM (n = 3). *, p<0.05. C) palladin, myocardin and MRTFs interdependently regulate SMC marker genes. SMA promoter luciferase construct was cotransfected with MRTF-A, -B, Myocardin, or palladin expressing plasmids into wild type, palladin null, and myocardin null APSCs. Luciferase activities were measured. Values represent mean ±SEM of three independent experiments. *, p<0.05 compared to WT.

The SRF cofactors, myocardin and MRTFs have been shown to strongly up-regulate a number of SMC marker genes [2], [9], [33], [37] in a CArG dependent manner. To determine if the effects of palladin on the SMC marker genes were dependent on these SRF co-activators, luciferase activity assays were conducted in A404 cells. A404 cells were co-transfected with palladin +/− dominant negative myocardin or MRTF A/B siRNAs, and the wild type SMA promoter luciferase construct. Suppression of myocardin and MRTFs decreased the basal SMA promoter activity in agreement with previous findings [31], [33]. Moreover, silencing of these factors partially attenuated the ability of palladin to enhance SMA promoter activity (Fig. 5A, p<0.05), in support of the hypothesis that effects of palladin involve interaction with these SRF co-activators. This hypothesis was further tested by comparing the activity of the SMA promoter luciferase construct in SMC derived from wild type and palladin KO ESCs [26]. Transgenic ESCs were generated that stably expressed a puromycin-resistance gene under the control of a SMA promoter. Negative selection was then used to purify SMCs from EBs. Purified SMCs expressing multiple SMC markers were designated APSCs (SMA-puromycin selected cells) [26]. In this model, the palladin null ESCs can still differentiate into SMCs based on the selection of SMA promoter. However, this population of cells has markedly decreased SMA, SM22, SM MHC, and calponin protein expression. In luciferase reporter assays, compared to the wild type APSCs, the basal level of SMA and SM22 promoter luciferase activity in palladin knock out APSCs was significantly decreased (p<0.05, Fig. 5B). The responses to myocardin, MRTFs, and palladin in both palladin and myocardin knock out APSCs were significantly decreased compared to that of wild type APSCs (Fig. 5C). These results suggest that palladin, myocardin and MRTFs are inter-dependent in regulation of SMC specific genes.

### The carboxyl terminus of palladin bound with MRTF

**Figure 6:**
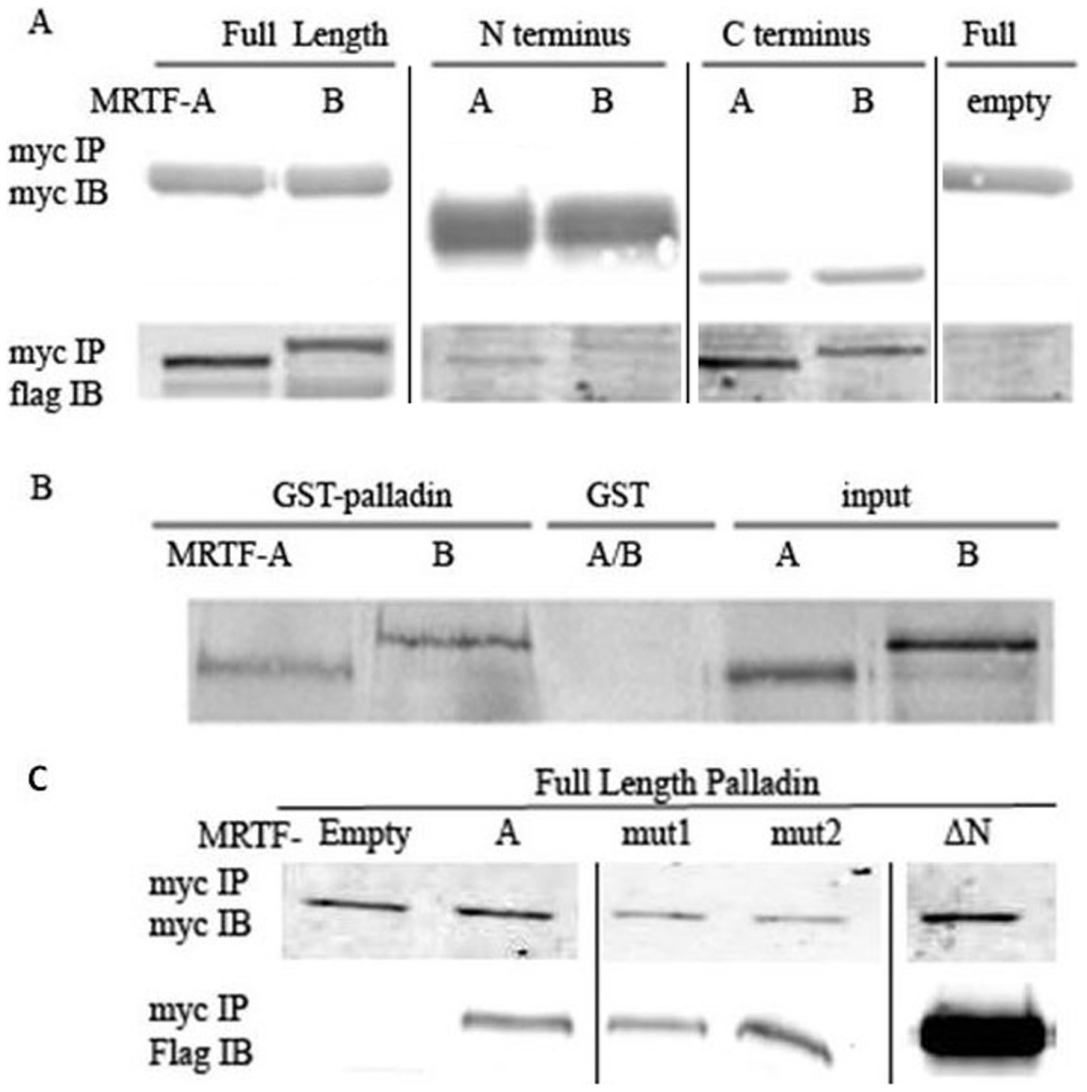
Carboxyl terminus of palladin interacts with MRTFs in SMCs. A) Full length, amino (N), or carboxyl (C) terminus of myc tagged palladin constructs were cotransfected with flag tagged MRTF-A or B into HEK-293 cells. Co-IP assays were performed, and proteins were detected with myc and flag antibodies. Note that different regions of the gels are shown for full length, and the N- and C-termini, which as expected, run differently, as shown in Fig. 2C. B) *in vitro* pull down assays showed that MRTF-A interacts with palladin directly. Recombinant GST tagged palladin was purified from E.Coli and MRTF-A and –B proteins were translated *in vitro*. C) MRTF-A interaction with palladin is not via the amino terminal RPEL domain. The two RPEL motifs (mut1: R33A and P34A and mut2: L39A and V40A) mutants and an amino terminal 100 aa deletion of MRTF-A (ΔN) were cotransfected with myc tagged palladin into HEK-293 cells, and co-IP was performed 36 h after transfection with anti-myc antibody and blotted with anti-myc or flag antibodies.

To determine whether the above results were caused by the direct interaction of MRTFs, myocardin, SRF and palladin proteins, co-immunoprecipitation (co-IP) assays were performed. Myc tagged palladin expression constructs were co-transfected with flag tagged MRTF-A or MRTF-B into HEK-293 cells. Cells were lysed 36 h after transfection, and co-IPs were performed with myc antibody. As shown in Fig. 6A, full length and the carboxyl-termini of palladin bound to both MRTF-A and –B. However, there was no detectable palladin interaction with myocardin and SRF under these experimental conditions (data not shown). The interaction of palladin with MRTF-A and –B was also confirmed with an *in vitro* GST pull down assay (Fig. 6B) using recombinant GST-palladin and *in vitro* translated MRTFs. The RPEL domain of in the amino-terminus of MRTF-A has been shown to be the critical motif for binding to G actin and for its transcriptional regulation [38]. We next tested whether the interaction of MRTF with palladin is via this motif. We mutated the RPEL motif or truncated its amino terminal 100 amino acids. The mutant MRTF-A and myc tagged palladin were co-transfected into HEK-293 cells, and IP was performed with myc antibody. Unexpectedly, as shown in Fig. 6C, the amino terminal deletion or the mutated RPEL of MRTF-A still bound to the full length of palladin, indicating that other MRTF-A motifs interact with palladin and that palladin does not compete with actin for the RPEL domain.

### Palladin localized to the nucleus in SMCs

**Figure 7:**
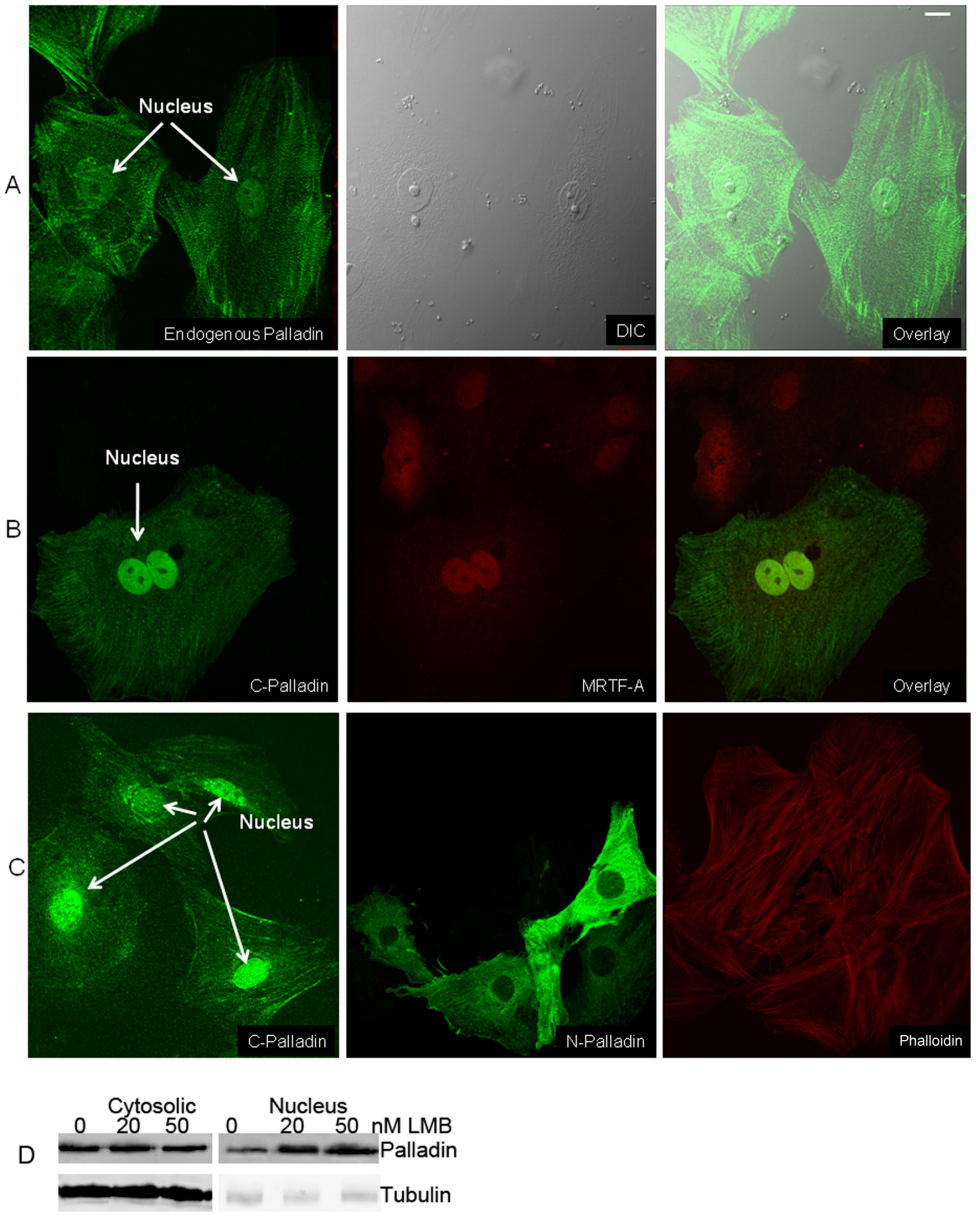
Confocal images demonstrating nuclear localization of full length endogenous palladin and of C terminal but not N terminal palladin. A) Endogenous palladin was detected in nuclei of mature SMCs (rat aortic SMCs). Cultured subconfluent R518 cells were fixed with cold methanol and stained with a palladin polyclonal antibody, and with Alexa fluor conjugated secondary anti-rabbit antibody. Inset shows the Western blot results of palladin in a nuclear fraction. B) Carboxyl (C) terminus of palladin co-localizes with MRTF-A in the nucleus. Rat aortic SMCs were transfected with myc tagged C-terminus of palladin. The C-terminus of palladin was detected with an anti-myc epitope antibody, and the endogenous MRTF-A was detected with an anti-MRTF antibody and secondary Alexa fluor conjugated anti-rabbit antibody. C) Expressed carboxyl (C) terminus but not amino (N) terminus of palladin localizes in the nucleus of R518 cells. Myc tagged N- or C- terminal palladin constructs were transfected into cultured R518 cells by electroporation. Cells were fixed after 72 h transfection, and proteins were detected with myc antibody. Secondary antibody labeling alone, under identical conditions showed no detectable fluorescence. Scale bar, 10 µm. D) endogenous palladin was accumulated in the nucleus by leptomycin B (LMB). Cultured subconfluent R518 cells were trypsinized and subjected to nucleus-cytosolic fractionation. Equal amounts of protein were loaded for Western bloting. Leptomycin B 20 and 50 nM increased the palladin expression level in the nucleus fraction. Tubulin was used as a marker for the cytosolic fraction.

In view of our finding that palladin can greatly induce the expression of SMC marker genes, we asked whether palladin shuttles to the nucleus to regulate downstream transcriptional activity. In order to confirm the role of palladin in differentiated SMCs, we studied the localization of palladin in rat aortic SMCs using immunofluorescence assays. We found that endogenous palladin can be detected in the nucleus of cultured rat aortic SMCs (Fig. 7A), A404 cells (data not shown), SMCs and endothelial cells in sections of mouse embryo E11.5 blood vessels, and human coronary vessels from patients with atherosclerotic disease (Figure S2). Nuclear localization of palladin also agrees with previously published results in kidney podocytes (7). Treatment with leptomycin B leads to a marked increase in endogenous palladin in the nuclear fraction (Fig. 7D). By overexpression of the amino and carboxyl terminus of palladin into rat aortic SMCs, we were able to shown that the carboxyl terminus of palladin localized in the nucleus, while the amino terminus of palladin localized in the cytoplasm along actin stress fibers (Fig. 7B). By immuno-localization assay, we also demonstrated that palladin and MRTF-A co-localize in the nucleus (Fig. 7C). The finding of nuclear palladin is consistent with its role in transcriptional regulation of SMC marker genes.

### Expression of SMC specific genes is attenuated in vessels during embryonic development in palladin null mice

**Figure 8:**
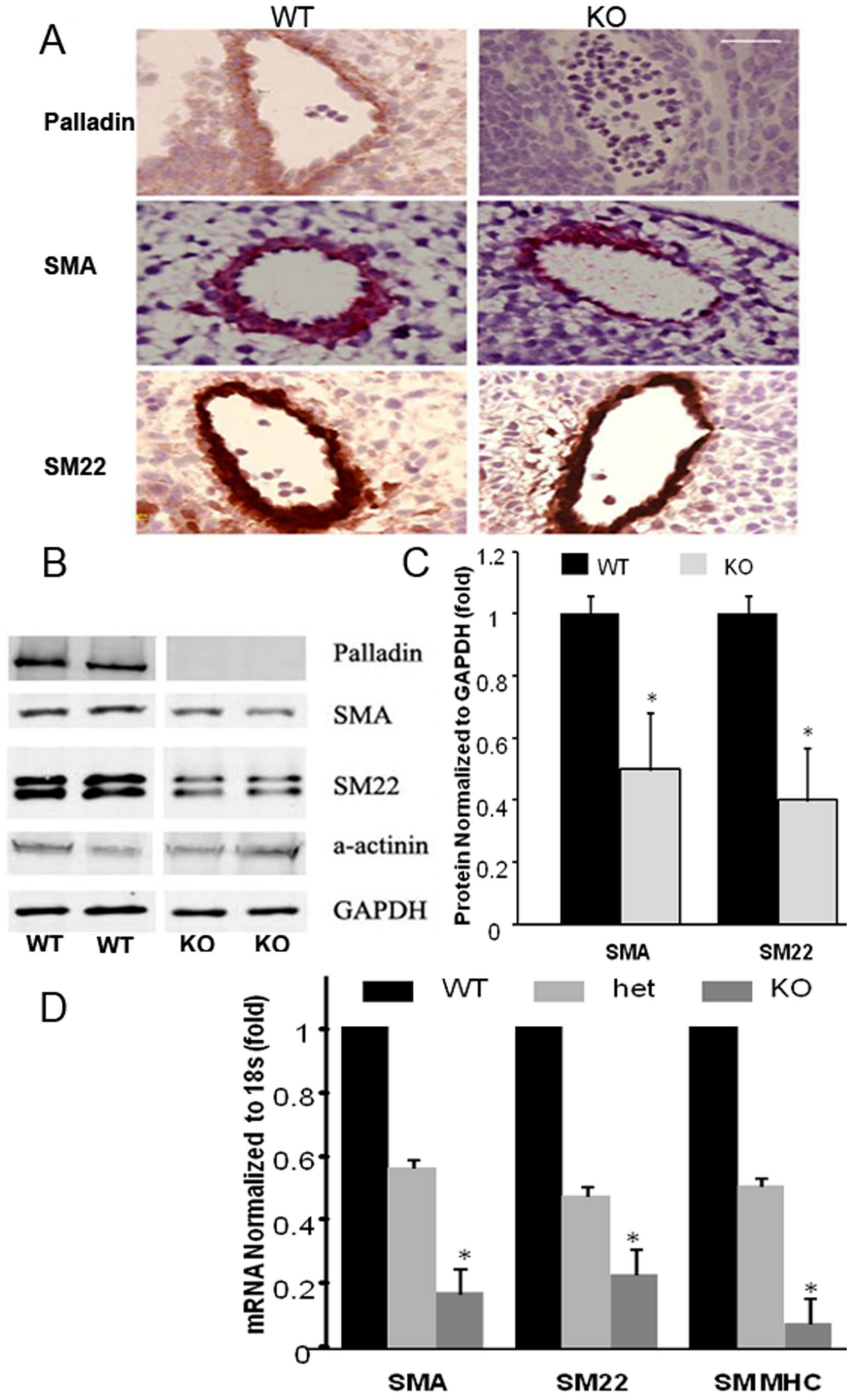
SMA and SM22 expression was attenuated in homogenates of palladin E11.5 knockdown embryos, in isolated umbilical vessels and in dorsal aortae. A) Representative images show that the expression of SMA and SM22 was attenuated in dorsal aortae at E11.5 embryos by immunohistochemistry analysis. Embryos were fixed and embedded for sectioning. Frontal sections were stained with palladin, SMA, and SM22 antibodies and corresponding secondary antibodies conjugated with biotin. The signals were developed with DAB and photographed on a Axioskop 2 Zeiss microscope. Scale bar, 50 µm. B) Western blots showing a significant decrease in the protein expression of SMA and SM22 in homogenates of whole embryos with its quantification shown in C. The whole embryos were dissected genotyped, and homogenized in RIPA buffer. An equal amount of protein was loaded for SDS-PAGE, and blotted with SMA, SM22, palladin, SMC specific α-actinin, and GAPDH antibodies. The signals were normalized to GAPDH. Values represent the mean±SEM (n = 3). *, p<0.05. D) The expression of SMA, SM22, and SM MHC mRNA is markedly decreased in umbilical vessels isolated from E11.5 palladin +/− and −/− mice compared to Wt. Umbilical vessels were dissected from E11.5 wt, het, and knockdown E11.5 embryos. RNA was extracted and SMA, SM22 and SM MHC quantitated by real time RT-PCR. Data was normalized to 18s. Values represent the mean±SEM (n = 3). *, p<0.05.

Palladin deficient mice die by day E15.5 and display multiple defects [22], indicating that palladin plays a critical role in embryonic development. The palladin null embryos are pale compared with wild type embryos suggesting poor circulation, an observation that has been reported previously and attributed to anemia [22]. However, the effects of KO of palladin on SMC have not been studied. The expression of SMA and SM22 was evaluated by immunohistochemistry in frontal sections cut through the dorsal aorta and associated great vessels of wild type and palladin null embryos. Neither the pattern of formation of the great vessels nor the size of the blood vessels in cross section showed a detectable difference in palladin null embryos compared to wild type (data not shown). As shown in Fig. 8A, the expression of SMA and SM22 was decreased in the dorsal aorta of palladin knockdown vessels at E11.5. Among the 13 embryos assessed for each group, there were no detectable differences in muscle cell layer width between wild type and palladin null embryos in vessel cross sections. Due to the difficulty in the quantification of staining intensity using immunohistochemistry, Western blotting was used to measure SMA and SM22 protein content in homogenates from whole embryos of palladin knockdown and wild type mice (Fig. 8B). There was approximately a 40–50% decrease of SMA and SM22 protein expression in palladin knockdown embryos compared to wild type (Fig. 8C). This evaluation was next extended specifically to blood vessels. We measured the expression of SMA and SM22 at the mRNA level in umbilical vessels isolated at E11.5. As shown in Fig. 8D, the mRNA expression of SMA, SM22 and SM MHC decreased by 70–80% in palladin knockdown vessels (p<0.05) and by 40–50% in vessels isolated from palladin heterozygous embryos. It was noted during dissection, that isolated umbilical vessels from wild type but not palladin null mice contracted during dissection, consistent with a difference in contractile protein content. These *in situ* results suggest that palladin plays a significant role in the induction of SMC differentiation during early embryonic development.

## Discussion

The actin associated protein, palladin, is a critical structural component of the actin cytoskeleton, and functions as a molecular scaffold interacting with multiple proteins in the actin cytoskeleton [21], [24], [39], [40], [41], [42]. Palladin exists as multiple isoforms, and the expression of different isoforms is regulated in a tissue specific manner, suggesting that different palladin variants may be specialized for different functions. Cells lacking palladin have disrupted actin organization and palladin null mice display defective neural tube and ventral closure leading to embryonic death [27]. Although it is clear that palladin is required for proper embryonic development, the role of palladin in the development and function of the vasculature or its mechanism of action in SMC is unknown. In the present study, we provide evidence suggesting that palladin plays an important role in the early stage of SMC differentiation. We found that palladin induced the expression of SMC marker genes *in vitro* using a SMC differentiation model; that over expression of palladin induced, while down regulation suppressed the expression of SMC marker genes; that palladin activated SMC promoter transcriptional activity through CArG elements by direct or indirect binding to the promoters of SMA or SM22; that palladin translocates and interacts with MRTFs in the nucleus; and that the transcriptional activities of palladin and MRTFs were inter-dependent. Importantly, in palladin deficient mice, the induction of the SM markers, SMA and SM22 was significantly attenuated at E 11.5 in whole embryos and in isolated blood vessels.

The expression of so-called SMC specific markers, such as SMA, SM22 and SM-MHC, is controlled by multiple transcription factors such as SRF and myocardin. Myocardin is exclusively expressed in SMCs and cardiomyocytes and potently induces the transcription of CArG containing SMC differentiation marker genes in the presence of SRF [37]. However myocardin does not appear to function in the early stage of induction of SMC differentiation genes, as myocardin null embryonic stem cells were able to differentiate into SMCs in the context of a chimeric knockout mouse [43], and the expression of myocardin unlike palladin was low in the early stages of development of vascular SMCs [37], [44]. These studies suggest that there may be alternative molecular mechanisms that contribute to the initiation of SMC differentiation. Palladin presents as early as E8.5 in mice embryos [45] and it is most highly expressed at 48 h following RA treatment in A404 (Fig. 1) and at 20 days in EB SMC differentiation models[26]. This precedes the induction of SMC marker genes. The induction of SMC marker transcriptional activity was most significant in undifferentiated A404 cells, whereas the enhancement is moderate in EB derived SMCs and adult aortic SMCs (A404>>>APSCs> = R518). In undifferentiated A404 cells, over-expression of palladin can greatly increase SMA and SM22 promoter activity 600 and 20 fold respectively, while in differentiated SMCs APSC and R518 cells the enhancement is much less. These results suggest that palladin may be a possible candidate in regulating early stage SMC differentiation. One possible mechanism for palladin regulation of expression of SMC marker genes is through MRTFs which are widely expressed, known to be important in regulating expression of SMC specific marker genes, and dependent on the Rho family GTPases and actin dynamics. Palladin's ability to increase the F:G actin would free MRTF from cytosolic G actin. In the luciferase reporter assays, the magnitude of the palladin-enhanced expression of SMC marker genes was markedly attenuated by down regulation of MRTFs and/or myocardin in cultured cells (Fig. 5A). This attenuation was greater when both MRTFs and myocardin were down regulated than when individually reduced. This suggests that palladin may function via a SRF-myocardin-MRTF pathway. By using pull down assays, we showed that the C-terminus of palladin directly interacts with the C-termini of MRTFs, which are important for transcriptional activation. This interaction was also confirmed by over-expression experiments that showed that the C-terminus of palladin co-localizes with MRTF-A in the nucleus of rat aortic SMCs and differentiated A404 cells. Palladin binding to MRTFs in the nucleus may promote chromatin remodeling and initiate the MRTF-SRF transcriptional activation of SMCs marker genes. In the reporter and over expression assays, both N-terminal and C-terminal halves of palladin are required for the expression of SMC genes. This plus the fact that the C-terminus of palladin localizes in the nucleus while the N-terminus localizes in the cytoplasm along stress fibers, suggests that nuclear and cytoplasmic distribution of palladin are both necessary for palladin's function. Whether palladin interacts with MRTFs in the cytoplasm and then translocates to nucleus, or they interact in the nucleus is not known.

**Figure 9:**
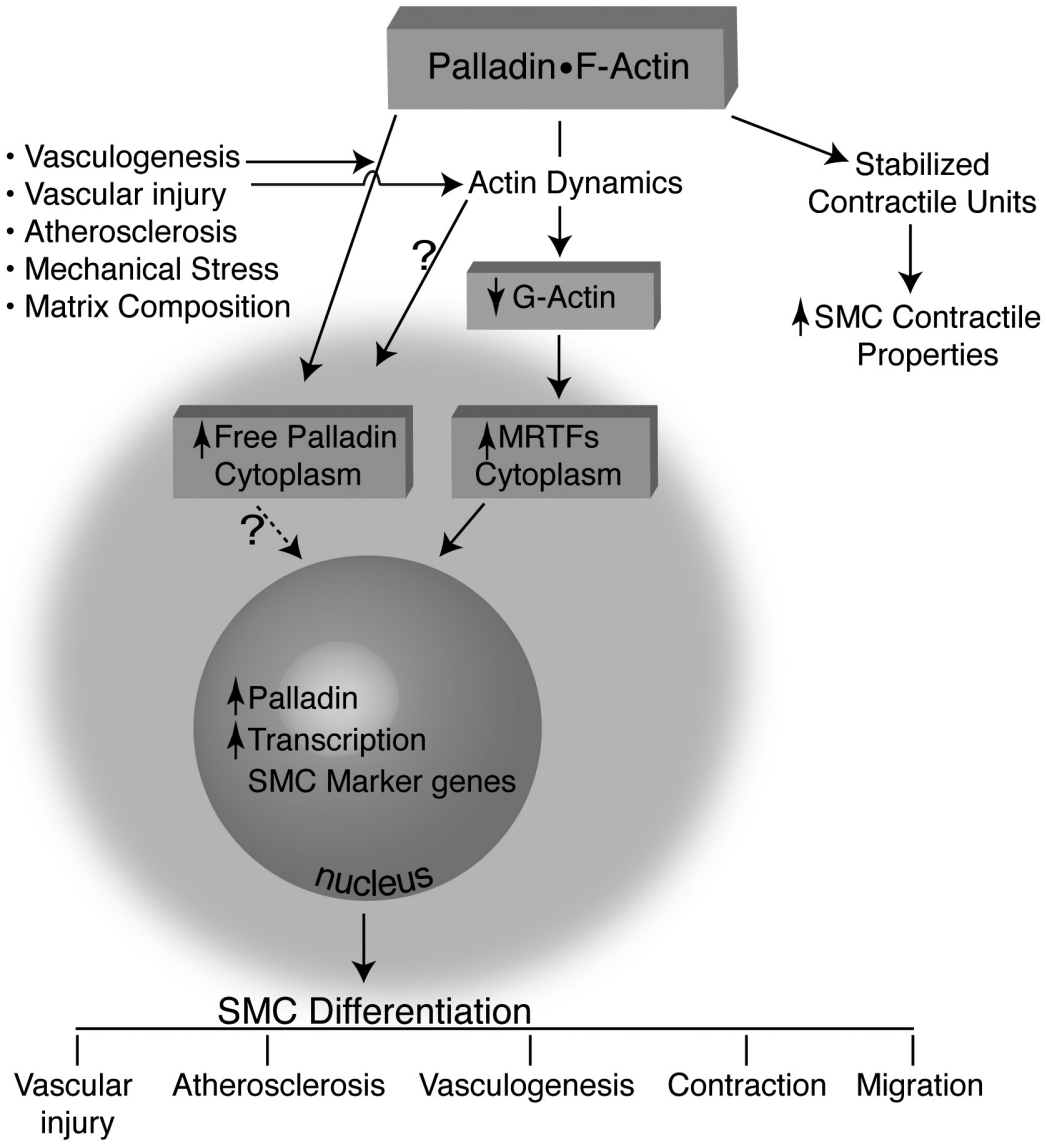
Scheme showing possible mechanisms whereby palladin regulates SMC differentiation. Palladin released from the cytoskeleton can translocate to the nucleus and regulate transcription either by binding directly to the promoters of SMC marker genes or by forming a complex with MRTFs and SRF or their associated proteins. In addition palladin's regulation of actin dynamics frees cytosolic MRTFs, from G actin which translocate to the nucleus to further enhance transcription of SMC marker genes.

Another possible mechanism is that palladin can directly interact with the promoters of SMC marker genes and initiate transcription of SMC marker genes. We showed that mutation of CArG elements in the SMC gene promoters significantly decreased the responses to palladin (Fig. 2). In addition, we found that palladin can bind to the SMA promoter within intact chromatin by ChIP assays (Fig. 4). Thus, it is also possible that palladin can shuttle to the nucleus and directly bind to SMC gene promoters to modulate chromatin structure and regulate transcription of SMC marker genes. We detected endogenous palladin in the nuclei of SMCs. We favor the hypothesis that palladin regulates SMC differentiation through both direct and indirect pathways. The possible mechanisms whereby palladin regulates SMC markers are illustrated in Fig. 9. On the one hand, palladin released from the cytoskeleton can translocate to the nucleus and regulate transcription either by binding directly to the promoters of SMC marker genes or by forming a complex with MRTFs and SRF to enhance transcription. In addition palladin's regulation of actin dynamics frees cytosolic MRTFs, which translocate to the nucleus to further enhance transcription of SMC marker genes.

Palladin deficient mice die by day E15.5 and display multiple defects including defective cranial neural tube closure and fetal liver herniation indicating that palladin plays a critical role in embryonic development. The exact mechanism whereby palladin knockout leads to embryonic lethality remains unclear and the importance of palladin in the development of the vasculature is not clear. However, results of the present studies provide clear evidence that palladin plays an important role in SMC differentiation in *in vitro* model systems. Moreover, consistent with these results, palladin knockdown embryos E11.5 showed decreased expression of SMA and SM22 protein in the dorsal aorta by immunohistochemstry analysis and in whole embryos by Western blotting (Fig. 8). Finally, a dramatic reduction in SMA, SMA and SM MHC mRNA was found in isolated umbilical vessels at E11.5 (Fig. 8). However, as no visible changes were observed in either the pattern or size of the great vessels or the umbilical vessels, palladin does not appear to contribute to the mechanisms underlying those processes at least at the E11.5 time point. In view of the partial expression of SMA and SM22 in the palladin knockdown vessels, palladin is either not absolutely required for their expression or compensatory mechanisms have been turned on. The decreased expression of the SMC markers at the protein level, in the palladin null embryos is consistent with our earlier finding of decreased force development in palladin null SMCs isolated from EBs. This repressed contractility of the vasculature likely contributes to the palladin null embryonic lethality due to the weakened vessel walls. The death of the embryos at about E15.5 corresponds to the time when the coronary circulation is perfused as it connects to the aorta.

Taken together, our findings demonstrate that palladin plays a key role, through both direct and indirect pathways, in the induction of SMC marker genes during the early stages of SMC differentiation. Further studies are needed to determine mechanisms by which palladin promotes transcriptional activation through binding to SMC promoters, and what genes may compensate, at least in part, for its loss.

## Supporting Information

Text S1 (0.02 MB DOCX)

Figure S1 Disruption of palladin in mice. A. Construct design for palladin gene disruption. See details in Experimental procedures. B. PCR analysis of genomic DNA from yolk sac of 10.5 dpc embryos derived from palladin+/− mouse intercrossing. C. Southern blot analysis. Several PCR-positive clonal DNA samples were digested with XhoI and NdeI and southern blotted. The detection of a 4.9 kb band indicates that the vector DNA has recombined at the correct site, introducing a novel XhoI site into the allele. D. Protein immunoblot analysis of mouse embryonic tissue from a heterozygote intercross. Palladin was identified with the monoclonal Ab 1E6 (1,2). WT, wild type; HET, heterozygote; KO, knockout. Same blot was reprobed with tubulin Ab for loading control.
(0.09 MB TIF)

Figure S2 Palladin localized to SMC and endothelial cell nuclei in human coronary vessels with atherosclerosis (upper panels) and in wild type but not palladin null mouse embryonic blood vessels (E11.5) (lower panels). Nuclear palladin was not present in all cells showing cytoplasmic palladin staining and may reflect different states of differentiation. Sections from fixed human coronary vessels and frontal sections from fixed and embedded embryos were stained with palladin antibody which one (we did not mention different antibodies in the text) and a anti-rabbit antibody conjugated with biotin. Signals were developed with DAB and photographed on a Axioskop 2 Zeiss microscope. scale bar: 10 µm.
(25.24 MB TIF)

## References

[1] Wang DZ, Li S, Hockemeyer D, Sutherland L, Wang Z (2002). Potentiation of serum response factor activity by a family of myocardin-related transcription factors.. Proc Natl Acad Sci U S A.

[2] Miralles F, Posern G, Zaromytidou AI, Treisman R (2003). Actin dynamics control SRF activity by regulation of its coactivator MAL.. Cell.

[3] Selvaraj A, Prywes R (2003). Megakaryoblastic leukemia-1/2, a transcriptional co-activator of serum response factor, is required for skeletal myogenic differentiation.. J Biol Chem.

[4] Liu ZP, Wang Z, Yanagisawa H, Olson EN (2005). Phenotypic modulation of smooth muscle cells through interaction of Foxo4 and myocardin.. Dev Cell.

[5] Wang Z, Wang DZ, Hockemeyer D, McAnally J, Nordheim A (2004). Myocardin and ternary complex factors compete for SRF to control smooth muscle gene expression.. Nature.

[6] Yoshida T, Gan Q, Owens GK (2008). Kruppel-like factor 4, Elk-1, and histone deacetylases cooperatively suppress smooth muscle cell differentiation markers in response to oxidized phospholipids.. Am J Physiol Cell Physiol.

[7] Owens GK, Kumar MS, Wamhoff BR (2004). Molecular regulation of vascular smooth muscle cell differentiation in development and disease.. Physiol Rev.

[8] Pipes GC, Creemers EE, Olson EN (2006). The myocardin family of transcriptional coactivators: versatile regulators of cell growth, migration, and myogenesis.. Genes Dev.

[9] Mack CP, Somlyo AV, Hautmann M, Somlyo AP, Owens GK (2001). Smooth muscle differentiation marker gene expression is regulated by RhoA-mediated actin polymerization.. J Biol Chem.

[10] Miano JM, Long X, Fujiwara K (2007). Serum response factor: master regulator of the actin cytoskeleton and contractile apparatus.. Am J Physiol Cell Physiol.

[11] Sotiropoulos A, Gineitis D, Copeland J, Treisman R (1999). Signal-regulated activation of serum response factor is mediated by changes in actin dynamics.. Cell.

[12] Vartiainen MK, Guettler S, Larijani B, Treisman R (2007). Nuclear actin regulates dynamic subcellular localization and activity of the SRF cofactor MAL.. Science.

[13] Otey CA, Rachlin A, Moza M, Arneman D, Carpen O (2005). The palladin/myotilin/myopalladin family of actin-associated scaffolds.. Int Rev Cytol.

[14] Parast MM, Otey CA (2000). Characterization of palladin, a novel protein localized to stress fibers and cell adhesions.. J Cell Biol.

[15] Liu XS, Luo HJ, Yang H, Wang L, Kong H (2007). Palladin regulates cell and extracellular matrix interaction through maintaining normal actin cytoskeleton architecture and stabilizing beta1-integrin.. J Cell Biochem.

[16] Boukhelifa M, Parast MM, Valtschanoff JG, LaMantia AS, Meeker RB (2001). A role for the cytoskeleton-associated protein palladin in neurite outgrowth.. Mol Biol Cell.

[17] Hwang SJ, Pagliardini S, Boukhelifa M, Parast MM, Otey CA (2001). Palladin is expressed preferentially in excitatory terminals in the rat central nervous system.. J Comp Neurol.

[18] Boukhelifa M, Hwang SJ, Valtschanoff JG, Meeker RB, Rustioni A (2003). A critical role for palladin in astrocyte morphology and response to injury.. Mol Cell Neurosci.

[19] Gamez J, Armstrong J, Shatunov A, Selva-O'Callaghan A, Dominguez-Oronoz R (2008). Generalized muscle pseudo-hypertrophy and stiffness associated with the myotilin Ser55Phe mutation: A novel myotilinopathy phenotype?. J Neurol Sci.

[20] Dixon RD, Arneman DK, Rachlin AS, Sundaresan NR, Costello MJ (2008). Palladin is an actin cross-linking protein that uses immunoglobulin-like domains to bind filamentous actin.. J Biol Chem.

[21] Goicoechea S, Arneman D, Disanza A, Garcia-Mata R, Scita G (2006). Palladin binds to Eps8 and enhances the formation of dorsal ruffles and podosomes in vascular smooth muscle cells.. J Cell Sci.

[22] Liu XS, Li XH, Wang Y, Shu RZ, Wang L (2007). Disruption of palladin leads to defects in definitive erythropoiesis by interfering with erythroblastic island formation in mouse fetal liver.. Blood.

[23] Rachlin AS, Otey CA (2006). Identification of palladin isoforms and characterization of an isoform-specific interaction between Lasp-1 and palladin.. J Cell Sci.

[24] Jin L, Kern MJ, Otey CA, Wamhoff BR, Somlyo AV (2007). Angiotensin II, focal adhesion kinase, and PRX1 enhance smooth muscle expression of lipoma preferred partner and its newly identified binding partner palladin to promote cell migration.. Circ Res.

[25] Wang HV, Moser M (2008). Comparative expression analysis of the murine palladin isoforms.. Dev Dyn.

[26] Jin L, Yoshida T, Ho R, Owens GK, Somlyo AV (2008). The actin associated protein palladin is required for development of normal contractile properties of smooth muscle cells derived from embryoid bodies.. J Biol Chem.

[27] Luo H, Liu X, Wang F, Huang Q, Shen S (2005). Disruption of palladin results in neural tube closure defects in mice.. Mol Cell Neurosci.

[28] Gorenne I, Jin L, Yoshida T, Sanders JM, Sarembock IJ (2006). LPP expression during in vitro smooth muscle differentiation and stent-induced vascular injury.. Circ Res.

[29] Sinha S, Wamhoff BR, Hoofnagle MH, Thomas J, Neppl RL (2006). Assessment of contractility of purified smooth muscle cells derived from embryonic stem cells.. Stem Cells.

[30] Yoshida T, Gan Q, Shang Y, Owens GK (2007). Platelet-derived growth factor-BB represses smooth muscle cell marker genes via changes in binding of MKL factors and histone deacetylases to their promoters.. Am J Physiol Cell Physiol.

[31] Yoshida T, Hoofnagle MH, Owens GK (2004). Myocardin and Prx1 contribute to angiotensin II-induced expression of smooth muscle alpha-actin.. Circ Res.

[32] Shang Y, Yoshida T, Amendt BA, Martin JF, Owens GK (2008). Pitx2 is functionally important in the early stages of vascular smooth muscle cell differentiation.. J Cell Biol.

[33] Yoshida T, Sinha S, Dandre F, Wamhoff BR, Hoofnagle MH (2003). Myocardin is a key regulator of CArG-dependent transcription of multiple smooth muscle marker genes.. Circ Res.

[34] Gorenne I, Nakamoto RK, Phelps CP, Beckerle MC, Somlyo AV (2003). LPP, a LIM protein highly expressed in smooth muscle.. Am J Physiol Cell Physiol.

[35] Jin L, Hastings NE, Blackman BR, Somlyo AV (2009). Mechanical properties of the extracellular matrix alter expression of smooth muscle protein LPP and its partner palladin; relationship to early atherosclerosis and vascular injury.. J Muscle Res Cell Motil.

[36] Manabe I, Owens GK (2001). Recruitment of serum response factor and hyperacetylation of histones at smooth muscle-specific regulatory regions during differentiation of a novel P19-derived in vitro smooth muscle differentiation system.. Circ Res.

[37] Du KL, Ip HS, Li J, Chen M, Dandre F (2003). Myocardin is a critical serum response factor cofactor in the transcriptional program regulating smooth muscle cell differentiation.. Mol Cell Biol.

[38] Mouilleron S, Guettler S, Langer CA, Treisman R, McDonald NQ (2008). Molecular basis for G-actin binding to RPEL motifs from the serum response factor coactivator MAL.. EMBO J.

[39] Ronty M, Taivainen A, Moza M, Otey CA, Carpen O (2004). Molecular analysis of the interaction between palladin and alpha-actinin.. FEBS Lett.

[40] Ronty MJ, Leivonen SK, Hinz B, Rachlin A, Otey CA (2006). Isoform-Specific Regulation of the Actin-Organizing Protein Palladin during TGF-beta1-Induced Myofibroblast Differentiation.. J Invest Dermatol.

[41] Boukhelifa M, Moza M, Johansson T, Rachlin A, Parast M (2006). The proline-rich protein palladin is a binding partner for profilin.. Febs J.

[42] Boukhelifa M, Parast MM, Bear JE, Gertler FB, Otey CA (2004). Palladin is a novel binding partner for Ena/VASP family members.. Cell Motil Cytoskeleton.

[43] Pipes GC, Sinha S, Qi X, Zhu CH, Gallardo TD (2005). Stem cells and their derivatives can bypass the requirement of myocardin for smooth muscle gene expression.. Dev Biol.

[44] Wang D, Chang PS, Wang Z, Sutherland L, Richardson JA (2001). Activation of cardiac gene expression by myocardin, a transcriptional cofactor for serum response factor.. Cell.

[45] Endlich N, Schordan E, Cohen CD, Kretzler M, Lewko B (2009). Palladin is a dynamic actin-associated protein in podocytes.. Kidney Int.

