# Supplementary material for: The Actin Associated Protein Palladin Is Important for the Early Smooth Muscle Cell Differentiation
Source: PLoS One. 2010 Sep 22;5(9):e12823. doi: 10.1371/journal.pone.0012823 (PMC2943901; doi:10.1371/journal.pone.0012823)
Supplement: Text S1 — (0.02 MB DOCX) [file pone.0012823.s001.docx]

**The Actin Associated Protein Palladin is Important for the Early Smooth Muscle Cell Differentiation**

Li Jin, Qiong Gan, Bartosz J. Zieba, Silvia M Goicoechea*, Gary K. Owens, Carol A. Otey*, Avril V. Somlyo

Department of Molecular Physiology and Biological Physics, University of Virginia, Charlottesville, VA 22908; * Department of Cell and Molecular Physiology, University of North Carolina at Chapel Hill, Chapel Hill, NC 27599

**Generation of palladin ^-^/^-^ mice**

The second coding exon of palladin's 90 kDa isoform was targeted for Cre-recombinase mediated deletion by flanking the exon with two LoxP recombination sequences. LoxP sequences were introduced by recombination in murine embryonic stem cells of the SvEV129 strain. A plasmid was constructed using the pOSfrt-loxP vector backbone, which contains both the MC1neo cassette for positive selection and the PGK-TK cassette for negative selection. A 'short arm' of intronic DNA was amplified by PCR from SvEV129 genomic DNA using the forward primer gtcgacacaaggtttgtaaaggctaaagttggcacat and the reverse primer gtttaaacaacagagggcactggaaagtttgtaaagag. A 'long arm' of intronic DNA was amplified by PCR from SvEv129 genomic DNA with the forward primer gctagcaattgggaacagttcatgctcctttaatgt and the reverse primer cacgtgcccagaacagaaattgaactccatgtaaga. The targeted exon was amplified by PCR from SvEv129 genomic DNA with the forward primer (comprising a loxP sequence) ctcgagataacttcgtatagcatacattatacgaagttatagttgaaatgagctgtggtcgttccattc and the reverse primer atcgatccctgtatgccggagatgctaagtgtca. The targeted exon with 5' loxP sequence was then ligated into XhoI and ClaI sites the pOSfrt-loxP vector , in between the NeoMC1 and PGK-TK cassettes. The 'long arm' was ligated into the NheI and PmlI sites, in between the targeted exon and the PGK-TK cassette. The short arm was ligated into the SalI and PmeI sites, downstream of the MC1Neo cassette. The completed vector was transfected by electroporation of stem cells. Cells incorporating the NeoMC1 cassette were selected for by supplementing the media with neomycin. To ensure site-specific recombination, cells containing the PGK-TK cassette, which falls outside the long and short arms of the plasmid, were selected against by supplementing with ganciclovir. Surviving colonies of ES cells were genotyped by PCR and southern blot to ensure that the targeted exon had properly recombined with the plasmid sequence. Positive colonies were then electroporated with FLPase vector to remove the MC1Neo cassette from the recombined allele by virtue of two 'frt' recombination sequences flanking this cassette. A mixed colony of cells, some with and some without the NeoMC1 cassette were injected into C57BL/6J blastocyts. These blastocycts were implanted into a surrogate C57BL/6J female. Resultant chimera mice transmitting the loxP conditional allele without the MC1Neo cassette were backcrossed to wildtype C57BL/6J for the creation of conditional palladin knockout lines in this strain. Chimeras transmitting the allele of palladin that is disrupted by the presence of the MC1Neo cassette were crossed with wild type SvEV129 mice to produce isogenic heterozygous +/- palladin mice. Homozygous null mice were created by crossing two MC1Neo-palladin heterozygotes.

1- Parast MM, Otey CA (2000) Characterization of palladin, a novel protein localized to stress fibers and cell adhesions. J Cell Biol 150: 643–656

2- Goicoechea SM, Bednarski B, Garcia-Mata R, Prentice-Dunn H, Kim HJ, et al. (2008) Palladin contributes to invasive motility in human breast cancer cells. Oncogene 28: 587–98.
