# Supplementary figures and images for: The Actin Associated Protein Palladin Is Important for the Early Smooth Muscle Cell Differentiation
Source: PLoS One. 2010 Sep 22;5(9):e12823. doi: 10.1371/journal.pone.0012823 (PMC2943901; doi:10.1371/journal.pone.0012823)

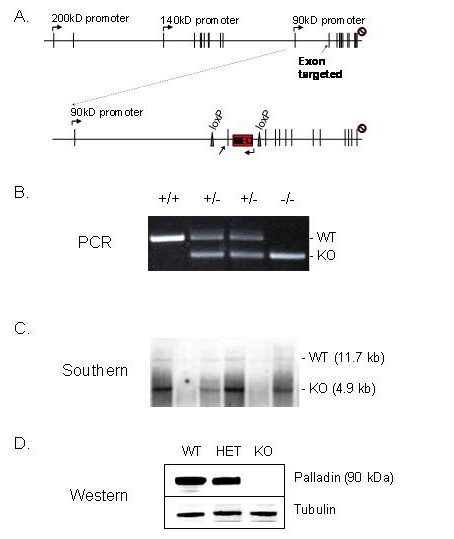

Supplement: Figure S1 — Disruption of palladin in mice. A. Construct design for palladin gene disruption. See details in Experimental procedures. B. PCR analysis of genomic DNA from yolk sac of 10.5 dpc embryos derived from palladin+/− mouse intercrossing. C. Southern blot analysis. Several PCR-positive clonal DNA samples were digested with XhoI and NdeI and southern blotted. The detection of a 4.9 kb band indicates that the vector DNA has recombined at the correct site, introducing a novel XhoI site into the allele. D. Protein immunoblot analysis of mouse embryonic tissue from a heterozygote intercross. Palladin was identified with the monoclonal Ab 1E6 (1,2). WT, wild type; HET, heterozygote; KO, knockout. Same blot was reprobed with tubulin Ab for loading control. (0.09 MB TIF) [file pone.0012823.s002.tif]

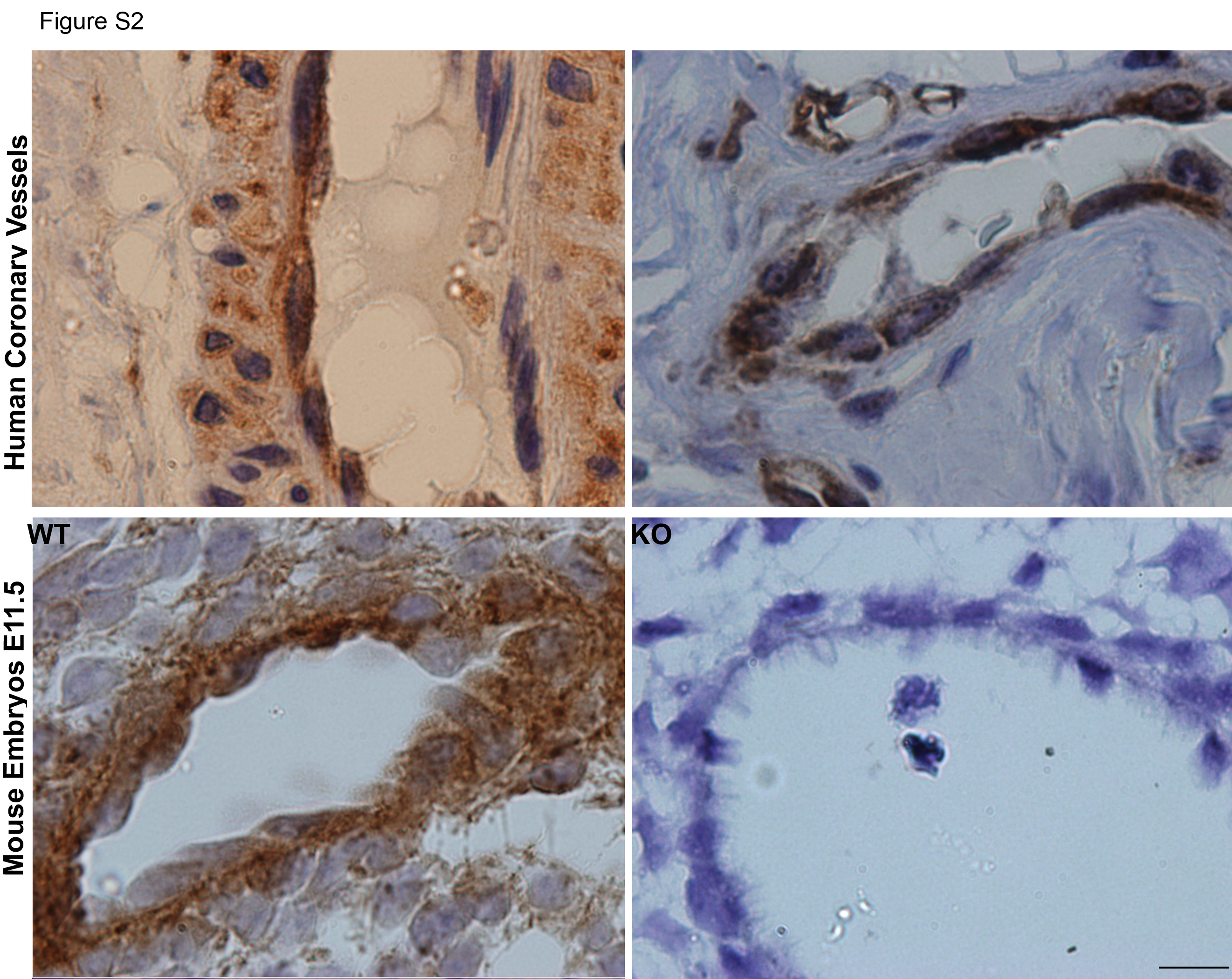

Supplement: Figure S2 — Palladin localized to SMC and endothelial cell nuclei in human coronary vessels with atherosclerosis (upper panels) and in wild type but not palladin null mouse embryonic blood vessels (E11.5) (lower panels). Nuclear palladin was not present in all cells showing cytoplasmic palladin staining and may reflect different states of differentiation. Sections from fixed human coronary vessels and frontal sections from fixed and embedded embryos were stained with palladin antibody which one (we did not mention different antibodies in the text) and a anti-rabbit antibody conjugated with biotin. Signals were developed with DAB and photographed on a Axioskop 2 Zeiss microscope. scale bar: 10 µm. (25.24 MB TIF) [file pone.0012823.s003.tif]
